# Alternative ground covers and strip-tilling in CBD hemp production

**DOI:** 10.1186/s13104-023-06551-4

**Published:** 2023-10-04

**Authors:** Erika Osorio, Benjamin Fisher, Matt Foster, Brian Voigt, Eric J. B. von Wettberg

**Affiliations:** 1 Department of Statistics, University of Vermont, Burlington, VT USA; 2 Four Suns Farm, Bridport, VT USA; 3 Department of Plant and Soil Science, University of Vermont, Burlington, VT 05405 USA; 4 Gund Institute for the Environment, University of Vermont, Burlington, VT 05405 USA

**Keywords:** Understory companion crops, Soil health, Black plastic, CBD Hemp, Ground cover, Strip-tilling, Carbon sequestration, Vermont, Experimental design, Hay, Clover, Forage crops

## Abstract

**Objective:**

Little research has been done on managing soil health for large-scale, outdoor hemp production, contributing to the possible overuse of black plastic for weed suppression. Our experiment aimed to understand the performance of alternative ground covers including forage crops and hay as well as a less disruptive tilling method called strip-tilling compared to black plastic.

**Results:**

Yield and soil health data were taken from three experimental plantings from two different outdoor CBD hemp farms in Vermont, USA. We find that hay may be a competitive alternative to black plastic in terms of producing heavier plants. Our research also found that clover seed and hay are both more cost-effective options than black plastic which may sway some farmers to adopt these alternative ground cover options.

## Introduction

In Vermont, a state in the Northeastern US, the acreage devoted to hemp production increased 228% from 2711 acres in October 2018 [1] to 8880 acres in October 2019 [2]. Due to past legal restrictions, little research has been conducted concerning best soil management practices for fields under hemp production. The rapid increase in acreage under hemp production combined with the lack of research on best soil health management practices may result in unnecessary runoff, soil nutrient loss, and overall soil degradation.

Of particular concern is the overuse of black plastic for suppressing weeds in large-scale outdoor hemp production. Alternatives to black plastic include understory legume companion crops like red clover, white clover, and fenugreek. Cover crops have the potential to suppress weeds, and may also increase soil nitrogen, sequester carbon, reduce soil erosion, and increase water retention [3]. Other sustainable weed suppression techniques include using hay as a ground cover or strip-tilling, a method where narrow strips are tilled into undisturbed vegetation or crop stubble.

Farmer adoption of sustainable weed suppression techniques will depend on efficiency and cost-effectiveness of the methods compared to black plastic. Research was conducted on experimental plantings of hemp using alternative ground covers, with the objective of determining if understory companion crops (red clover, white clover, fenugreek, blends), hay, and strip-tilling can result in equal or better yields, provide soil nitrogen, and sequester carbon at a price comparable to black plastic.

## Main text

### Data description

**Fig. 1:**
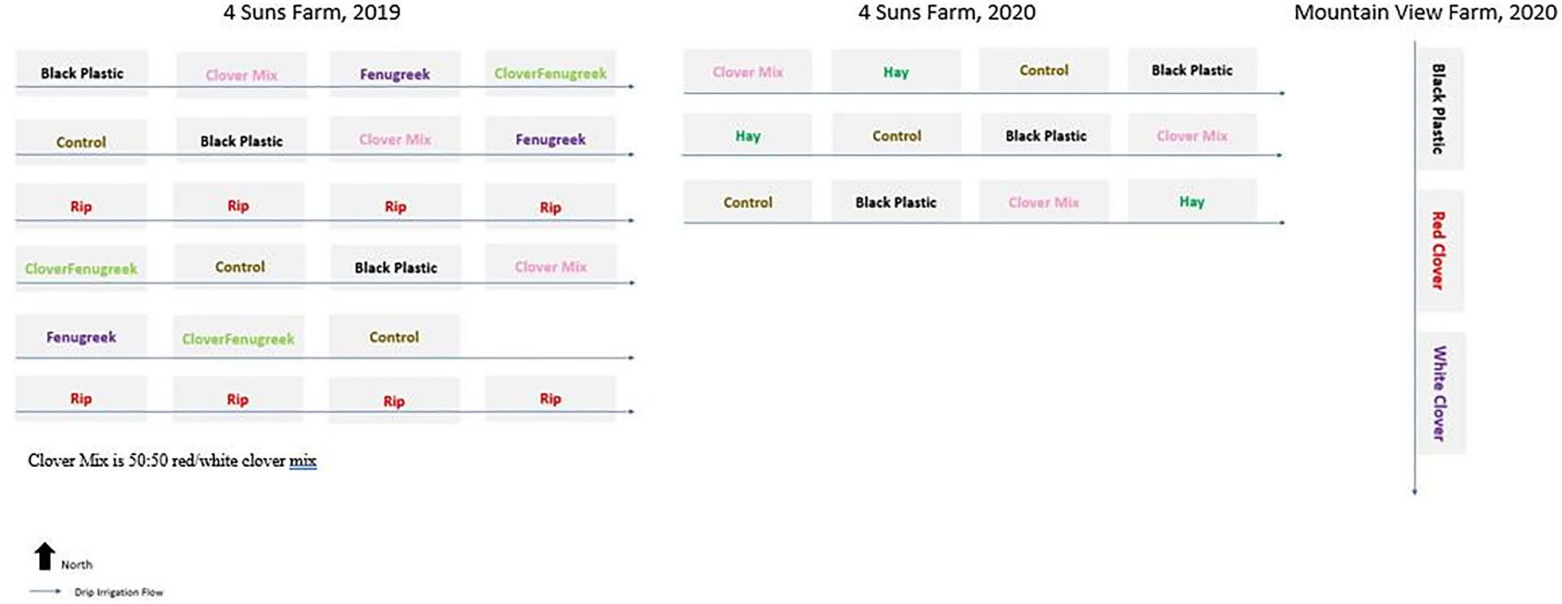
Treatment layout for each farm and year

The experimental plantings were conducted on two separate farms: 4 Suns Farm in Bridport, VT, in 2019 and 2020; and Mountain View Farm in Waitsfield, VT, in 2020. The differences in farm soil composition, year, hemp variety, treatments, and dependent variables resulted in three different experimental designs (Fig. 1) and five different data sets. All beds in each experiment design were rototilled except for the strip-tilled beds in which two-inch-wide strips were tilled into the present vegetation which consisted of mainly pasture forage.

#### 2019, 4 Suns Farm

Due to the heavy machinery necessary for strip-tilling, all treatments except for strip-tilling were randomly assigned to the beds. Twelve plants were planted per bed, spaced three feet apart in rows. To save the farmer money, only three plants were randomly sampled per replication, except for the clover/fenugreek mix. The plants were measured on flower bud weight and stem weight, both in grams.

#### 2020, 4 Suns Farm

Twelve plants were planted per bed with three-foot spacing between rows. Due to plant establishment issues, all plants could not be measured, resulting in unequal sample sizes per treatment. The treatments were not randomized due to farm logistics. Measurements of height (in) and wet weight (lbs.) were taken to quantify yield. Of the plants measured for harvest height and wet weight, a random sample of three plants per treatment were measured for flower bud weight (g) and stem weight (g). One soil sample was collected from each replication for each treatment.

#### 2020, Mountain View Farm

The experiment design tested three treatments with only one replication of each, resulting in pseudoreplication. Unfortunately, no yield data was provided for this location, however, three soil samples were taken per bed.

### Methods and results

Prior to analysis, all five data sets were checked for missing values, extreme outliers, and violated assumptions for MANOVA. Bonferroni corrections were applied with a significance acceptance criterion of p < 0.025 to ANOVA tests.

#### 2019, 4 Suns Farm yield data [flower weight (g), stem weight (g)]

Due to the multicollinearity between flower weight and stem weight, the greater statistical power of MANOVA, which comes from the correlation structure, cannot be used here. Therefore, Welch ANOVA tests were conducted for both variables. The Welch ANOVAs tested ${H}_{0}: {\mu }_{Black Plastic}={\mu }_{Control}={\mu }_{Clover Mix}={\mu }_{Clover/Fenugreek}={\mu }_{Fenugreek}={\mu }_{Strip-till}$ for flower weight and stem weight individually. The Welch ANOVAs for both variables were rejected with F statistic = 5.44 and p-value = 0.003 for flower weight and F statistic = 4.18 and p-value = 0.01 for stem weight. Thus, we can conclude that at least two treatment means are significantly different from each other with respect to one dependent variable. Following a Welch ANOVA, Games Howell pairwise comparisons resulted in one significant difference in flower weight where hemp grown in the clover mix was heavier than the flower weight for hemp grown in the rip treatment (p = 0.034).

#### 2020, 4 Suns Farm yield data [height at harvest (in), wet weight at harvest (lbs.)]

MANOVA tested ${H}_{0}:{{\varvec{\mu}}}_{Black Plastic}={{\varvec{\mu}}}_{Clover Mix}={{\varvec{\mu}}}_{Control}={{\varvec{\mu}}}_{Hay}$ and was rejected with p < 0.001. Post hoc ANOVAs tested ${H}_{0}:{\mu }_{Black Plastic}={\mu }_{Clover Mix}={\mu }_{Control}={\mu }_{Hay}$ for each dependent variable. We failed to reject for the height variable with F statistic = 2.13 and p-value = 0.101 and reject for wet weight with F statistic = 12.2 and p-value < 0.001. Thus, the treatment group means are all equal with respect to height and that at least two treatment means are significantly different from each other with respect to wet weight. Following ANOVA, Tukey’s HSD pairwise comparisons found that hemp grown in black plastic was significantly, although marginal, heavier than the hemp grown under the clover mix treatment (Table 1). Hemp grown under the hay treatment was significantly heavier than the hemp grown under the other three treatments (Table 1).

**Table 1:** Tukey HSD pairwise comparison results for wet weight, 2020 4 Suns Farm

Pairwise Comparisons Treatment 1–Treatment 2	Estimate	Conf.low	Conf.high	p.adj
Wet weight (lbs.)				
Clover mix- black plastic	− 1.748	− 3.484	− 0.012	0.048*
Control-black plastic	− 0.183	− 1.919	1.552	0.993
Hay-black plastic	2.102	0.326	3.877	0.013*
Control-clover mix	1.564	− 0.057	3.186	0.063
Hay-clover mix	3.850	2.186	5.514	< 0.001***
Hay-control	2.285	0.621	3.950	0.003**

Treatment 1 mean–Treatment 2 mean = estimate

*P < 0.05, **P < 0.01, and ***P < 0.001

#### 2020, 4 Suns Farm yield data [flower weight(g), stem weight (g)]

Due to the non-significant correlation coefficient between flower weight and stem weight we cannot take advantage of MANOVA’s benefits, thus, individual ANOVAs for each variable would be best. ANOVAs tested ${H}_{0}:{\mu }_{Black Plastic}={\mu }_{Clover Mix}={\mu }_{Control}={\mu }_{Hay}$. We failed to reject for both variables with F statistic = 0.296 and p-value = 0.827 for flower weight and F statistic = 2.058 and p-value = 0.184 for stem weight. Thus, we conclude that the treatment group means are all equal with respect to both flower weight and stem weight.

#### 2020, 4 Suns Farm soil health data [total nitrogen (grams total N/ grams dry soil), active carbon (PPM)]

Although the Pearson’s correlation between total nitrogen and active carbon is 0.56 we fail to reject a test for ${H}_{0}: \rho =0$ with p = 0.06. Thus, we are unable to take advantage of MANOVA and performed individual ANOVAs for each variable instead. ANOVAs tested ${H}_{0}:{\mu }_{Black Plastic}={\mu }_{Clover Mix}={\mu }_{Control}={\mu }_{Hay}$. We failed to reject for both variables with F statistic = 1.469 and p-value = 0.294 for total nitrogen and F statistic = 1.529 and p-value = 0.28 for active carbon. Therefore, we conclude that the treatment group means are all equal with respect to both total nitrogen and active carbon.

#### 2020, Mountain View Farm soil health data [total nitrogen (grams total N/grams dry soil), active carbon (PPM)]

Again, there is a non-significant correlation coefficient between total nitrogen and active carbon thus ANOVAs were performed for each variable. ANOVAs tested ${H}_{0}:{\mu }_{Black Plastic}={\mu }_{Red Clover}={\mu }_{White Clover}$. We failed to reject for both variables with F statistic = 0.138 and p-value = 0.874 for total nitrogen and F statistic = 1.139 and p-value = 0.3810 for active carbon. Thus, the treatment group means are all equal with respect to both total nitrogen and active carbon.

#### Price comparisons

Black plastic was compared to clover seed in terms of cost per acre, labor required, and soil benefits. Using the prices in Table 2, the total cost for three rolls of 4 × 4000 ft black plastic would range from $327 to $762. For most clover seed at most 20 lbs. per acre would be needed ranging between $112-$360 for 20 pounds of seed. A disposal fee for the black plastic will be charged at the landfill each year, while clover seed may only need to be acquired in smaller quantities after the first planting to replenish beds, making it a competitive option.

**Table 2:** Vendor, seeding/sizing, and price information for different clover based cover crops and black plastic

Cover crop	Vendor	Lbs./Acre	Price
Hemp mix cover crop	High mowing seeds	20	5 lb-$60 20 lb-$164
Medium red clover	High mowing seeds	20	1 lb-$10 5 lb-$37.75 20 lb-$112 40 lb-$197
White clover	High mowing seeds	5–9 drilled 7–14 broadcast	1 lb-$25 5 lb-$95 20 lb-$360 40 lb-$583.40
Medium red clover	Johnny’s selected seeds	5–15	1 lb-$12.50 5 lb-$49.10 25 lb-$155.25 50 lb-$290.50
New Zealand white clover	Johnny’s selected seeds	10	1 lb-$11.05 5 lb-$44.00 25 lb-$155.25 50 lb-$290.50

Black plastic	Vendor	Sizing(1 mil)	Price
Embossed black mulch	Johnny’s selected seeds	4 × 600 4 × 2000 4 × 4000	$70.75 $134 $254
Embossed black mulch	Nolt’s produce	4 × 2400 4 × 4000	$67 $109

Selection and pricing from April, 2021

In terms of labor required, using a tractor will most likely only require one person to lay plastic or to broadcast seed. Plastic mulch layer attachments for tractors cost around $2000, while broadcast seeder attachments for tractors can cost between $500 and $1000. Manual installation of black plastic is best done with at least two people. Black plastic will also need to be remove by hand or with a tractor. The undercutter tractor attachment needed to loosen the black plastic for removal can cost around $1000. Manual broadcast seeding typically requires one person, depending on the size of the plot.

Hay also seems to be a competitive contender compared to black plastic. For many farmers with large plots, hay is free or can be easily sourced from an adjacent farm for little cost. Hay is biodegradable, adds organic matter to soil, aids in water retention, and the process of growing hay sequesters carbon. However, it may be labor intensive to cut, dry, and lay the hay.

### Discussion

The 2019 4 Suns data on flower bud weight and stem weight resulted in one significant pairwise comparison where the flower weight of hemp grown in the clover mix treatment was heavier than that of hemp grown in the strip-tilled beds (p = 0.034). The 2020 4 Suns data on flower bud weight and stem weight resulted in all treatments being equal for both variables. It is possible that the small sample size did not provide enough power to detect differences. Using pairwise comparisons, the 2020 4 Suns harvest height and wet weight data resulted in hemp grown in the hay treatment being significantly heavier than hemp grown in all other treatments (black plastic, clover mix, control). The same analysis also showed that hemp grown in black plastic was marginally heavier than hemp grown in the clover mix treatment (p = 0.0477). However, due to the non-randomization of the experimental design the results may be biased by some lurking variable.

Both 2020 soil health data sets for 4 Suns Farm and Mountain View Farm resulted in no significant differences between treatments for total nitrogen and active carbon. The 2020 Mountain View Farm experiment design was pseudoreplicated and since three samples were taken from each treatment bed the analysis says more about the accuracy of the soil testing methods than about the differences between the beds. The lack of significance most likely stems from the fact that the fields were already high in nitrogen and active carbon, so any differences were most likely overshadowed by the already fertile soils. It is also possible that changes in total nitrogen or active carbon are not detectable in a single-season experiment. Lastly, the small sample sizes most likely do not give us enough power to detect any potential differences in total nitrogen and active carbon.

## Limitations

The 2020 4 Suns Farm experiment design was a non-randomized design and was not based on any blocking factor. Randomization allows for uncontrollable factors to be distributed equally such as field gradient, soil type, soil moisture, or other hidden spatial variation and thus acts as insurance against bias from an unknown variable [4]. Thus, the treatment means in the 2020 4 Suns Farm outcome variables may not differ from their true values only by random variation resulting in possibly biased results [5]. The pseudoreplication in the 2020 Mountain View Farm design also influences the validity of inference because there is no way to differentiate between the effect of the bed and the effect of the treatment. Thus, bed location and treatment are confounded, and the variability will be underestimated [6].

It is highly probable that the experiment also suffered from insufficient power to detect differences due to small sample sizes for some datasets. It is not surprising that data sets with small group sample sizes (three observations) resulted in non-significant results and thus it is very likely we have committed type II errors. Future studies should conduct a power analysis prior to designing the experiment ensuring proper sample sizes.

## Data Availability

Data is fully available without restrictions from the Open Science Foundation: https://osf.io/gdpfn/?view_only=630b45e49ba64fb585f4c1aefe398d1b

## Abbreviations

CBD: Cannabidiol
in: Inches
g: Grams
lbs.: Pounds
MANOVA: Multivariate analysis of variance
ANOVA: Analysis of variance
